# Effect of Dietary Fibers on Cecal Microbiota and Intestinal Tumorigenesis in Azoxymethane Treated A/J Min/+ Mice

**DOI:** 10.1371/journal.pone.0155402

**Published:** 2016-05-19

**Authors:** Birgitte Moen, Kristi Henjum, Ingrid Måge, Svein Halvor Knutsen, Ida Rud, Ragna Bogen Hetland, Jan Erik Paulsen

**Affiliations:** 1 Nofima - Norwegian Institute of Food, Fisheries and Aquaculture Research, Ås, Norway; 2 Department of Pharmacology, Oslo University and Oslo University Hospital, Oslo, Norway; 3 Department of Food, Water and Cosmetics, Division of Environmental Medicine, Norwegian Institute of Public Health, Oslo, Norway; 4 Norwegian University of Life Sciences, Department of Food Safety and Infection Biology, Oslo Norway; University of Illinois at Chicago, UNITED STATES

## Abstract

Foods naturally high in dietary fiber are generally considered to protect against development of colorectal cancer (CRC). However, the intrinsic effect of dietary fiber on intestinal carcinogenesis is unclear. We used azoxymethane (AOM) treated A/J Min/+ mice, which developed a significantly higher tumor load in the colon than in the small intestine, to compare the effects of dietary inulin (IN), cellulose (CE) or brewers spent grain (BSG) on intestinal tumorigenesis and cecal microbiota. Each fiber was tested at two dose levels, 5% and 15% (w/w) content of the AIN-93M diet. The microbiota was investigated by next-generation sequencing of the 16S rRNA gene (V4). We found that mice fed IN had approximately 50% lower colonic tumor load than mice fed CE or BSG (p<0.001). Surprisingly, all three types of fiber caused a dose dependent increase of colonic tumor load (p<0.001). The small intestinal tumor load was not affected by the dietary fiber interventions. Mice fed IN had a lower bacterial diversity than mice fed CE or BSG. The *Bacteroidetes/Firmicutes* ratio was significantly (p = 0.003) different between the three fiber diets with a higher mean value in IN fed mice compared with BSG and CE. We also found a relation between microbiota and the colonic tumor load, where many of the operational taxonomic units (OTUs) related to low tumor load were significantly enriched in mice fed IN. Among the OTUs related to low tumor load were bacteria affiliated with the *Bacteroides* genus. These results suggest that type of dietary fiber may play a role in the development of CRC, and that the suppressive effect of IN on colonic tumorigenesis is associated with profound changes in the cecal microbiota profile.

## Introduction

Colorectal cancer (CRC) is the third most common cause of cancer mortality in the world [1, 2]. By far the majority of cases occur in developed countries (http://globocan.iarc.fr/Pages/fact_sheets_cancer.aspx), connecting CRC development to western diet (and lifestyle) [2]. Typical for the “Western Diet” is low consumption of foods naturally rich in dietary fiber as fruit and vegetables. Epidemiological studies have found a negative association between intake of foods naturally rich in dietary fiber and CRC development [3–6]. Furthermore, the World Cancer Research Fund and American Institute for Cancer Research judge data for foods containing dietary fiber to be convincing to reduce CRC risk [7]. However, it is unclear whether fiber as an isolated component protects against intestinal tumorigenesis since studies have shown both protective as well as aggregative effects on CRC development [8].

Dietary fiber was traditionally defined as the portions of plant foods resistant to digestion by human digestive enzymes; including polysaccharides and related substances such as lignin and phenolics [9]. This definition has been expanded to also include resistant starches and oligosaccharides, such as fructo- and galacto oligosaccharides (FOS, GOS), the first being a structural element of inulin [10, 11]. Hence a range of carbohydrates with different physicochemical properties (i.e. solubility and viscosity) is encompassed by the fiber definition. Fibers can be classified as soluble, as inulin or beta-glucan, or insoluble as the traditional cell wall materials e.g. cellulose. If fibers protect against CRC, the mechanisms for soluble and insoluble fibers are anticipated to be different. Insoluble fibers are only partly fermented [12] and bulks luminal contents and speed colonic transit and may thereby minimize the exposure time of the colonic epithelium to ingested carcinogens. Soluble fibers are generally readily fermented by bacteria in the lumen of the colon into short chain fatty acids (SCFAs), such as acetate, propionate and butyrate, and other metabolites with potentially beneficial properties [13]. Compared with other complex polysaccharide based fiber from fruits/vegetables and cereals, it is reasonable to suggest that inulin have a minimal influence on the physicochemical environment of the small intestine, but rather induce indirect effects through stimulation of certain bacteria in the colon [14–16].

Recent advancements in our knowledge of the human microbiota have shown that it exerts an important influence on human health. The greatest exposure to microorganisms occurs in the gut, particularly the colon, and accumulating data suggest that the microbiota has a role in the aetiology of several types of cancer by influencing inflammation, DNA damage and apoptosis [17, 18]. It has become increasingly clear that the activities of the gut microbiota, particularly their metabolites, strongly influence protection against, and predisposition to, the development of CRC [19, 20]. A recent study in gnotobiotic mice strongly support the hypothesis that fiber protects against CRC in a butyrate-dependent manner by influencing the microbiota [21].

The multiple intestinal neoplasia (Min/+) mouse is one of the most widely used murine models for human familial adenomatous polyposis (FAP). The Min/+ mouse is heterozygous for a mutation in the tumor suppressor gene *Apc*, analogous to the mutation seen in the human *APC* gene. This germline mutation leads to the development of numerous neoplastic intestinal polyps [22, 23]. Complete somatic inactivation of *Apc* in discrete crypts of the intestinal epithelium seems to be the initial event of the tumorigenesis in Min/+ mice, human FAP and in 80% of sporadic CRC in humans [24]. Contradictory to the human pathology, conventional C57 BL/6J Min/+ mice develop tumors predominantly in the small intestine [25–28]. This is a considerable drawback when colon specific factors, such as luminal microbiota, are studied. The novel Min/+ mouse on the A/J genetic background provides a better model for colon cancer as these mice spontaneously develop a considerable number of colonic adenomas that eventually progress to carcinomas in old individuals [29]. A/J Min/+ mice are also more susceptible to AOM-induced colon carcinogenesis than C57 BL/6J Min/+ mice [30]. Importantly, somatic inactivation of *Apc* seems to be the main mechanism also for the AOM-induced colonic tumorigenesis in Min/+ mice [31], as in untreated Min/+ mice thus modelling human carcinogenesis. The differential strain-dependent susceptibility to AOM between A/J and BL/6J was originally described in wild type mice [32], and thereafter three loci regulating differential response to AOM-induced colon carcinogenesis in these strains, were identified [33, 34].

In the present study we used AOM-treated A/J Min/+ mice, where the majority of tumors develops in the colon, to compare the effects of dietary inulin (IN), cellulose (CE) or brewers spent grain (BSG) on intestinal tumorigenesis and cecal microbiota. Cecal contents were chosen in this study as fermentation of indigestible food in mice, is compartmentalized in the cecum. Although the microbiota may vary between the cecum and the colon (and feces) studies have shown that the bacterial communities in the large intestinal and fecal samples cluster together and share a common “core” microbiota [35]. A recent study has also shown that the colonic-cecal contents and feces in the mouse have very similar metabolite profiles [36]. To profile the microbiota, next generation 16S rRNA gene sequencing (Illumina) of cecum content was performed.

The aim of this work was to i) investigate whether dietary intervention with IN, CE or BSG could influence intestinal tumorigenesis; ii) characterize the cecal microbiota in mice fed IN, CE or BSG; iii) explore potential association between microbiota and intestinal tumorigenesis.

## Material and Methods

### Mouse housing

All mice were bred at the animal facility of the National Institute of Public health (NIPH) (originally purchased from the Jackson Laboratory, Bar Harbour ME). A/J Min/+ mice were obtained by transferring the Apc Min/+ trait from C57Bl/6J Min mice to A/J mice, and backcrossing for more than 12 generations at the institute. A/J Min/+ males were mated with A/J wt females. Ear cartilage was used to extract DNA and the genotype was determined by use of allele-specific polymerase chain reaction (PCR) as described previously [37]. After weaning (at 3 weeks of age), siblings of the same sex were housed in the same individual ventilated cage, in a room with a 12 hours light/dark cycle and set temperature (19–23°C) and humidity (35–75%). Water and feed were given ad libitum. Harlan Teklad Extruded 2018 (Harlan Teklad, UK) was used as breeding diet during gestation and until weaning. Experimental diets were maintenance diets (see below/treatment). Sentinel animals were positioned in the rack. This study was approved by the Norwegian Animal Research Authority (NARA) (permit id 2379).

### Tumor induction

Azoxymethane (AOM) (Sigma-Aldrich, Oslo, Norway) was diluted in 0.9% NaCl. All pups were subcutaneously (s.c) injected with 8 mg/AOM/ kg body weight first at age 7 ±1 days and secondly at 14 ±1 days, always keeping seven days between the two injections. All injections were made in the morning (between 8 and 11 a.m) to avoid possible differences in AOM metabolism.

### Dietary treatment

**Fig 1:**
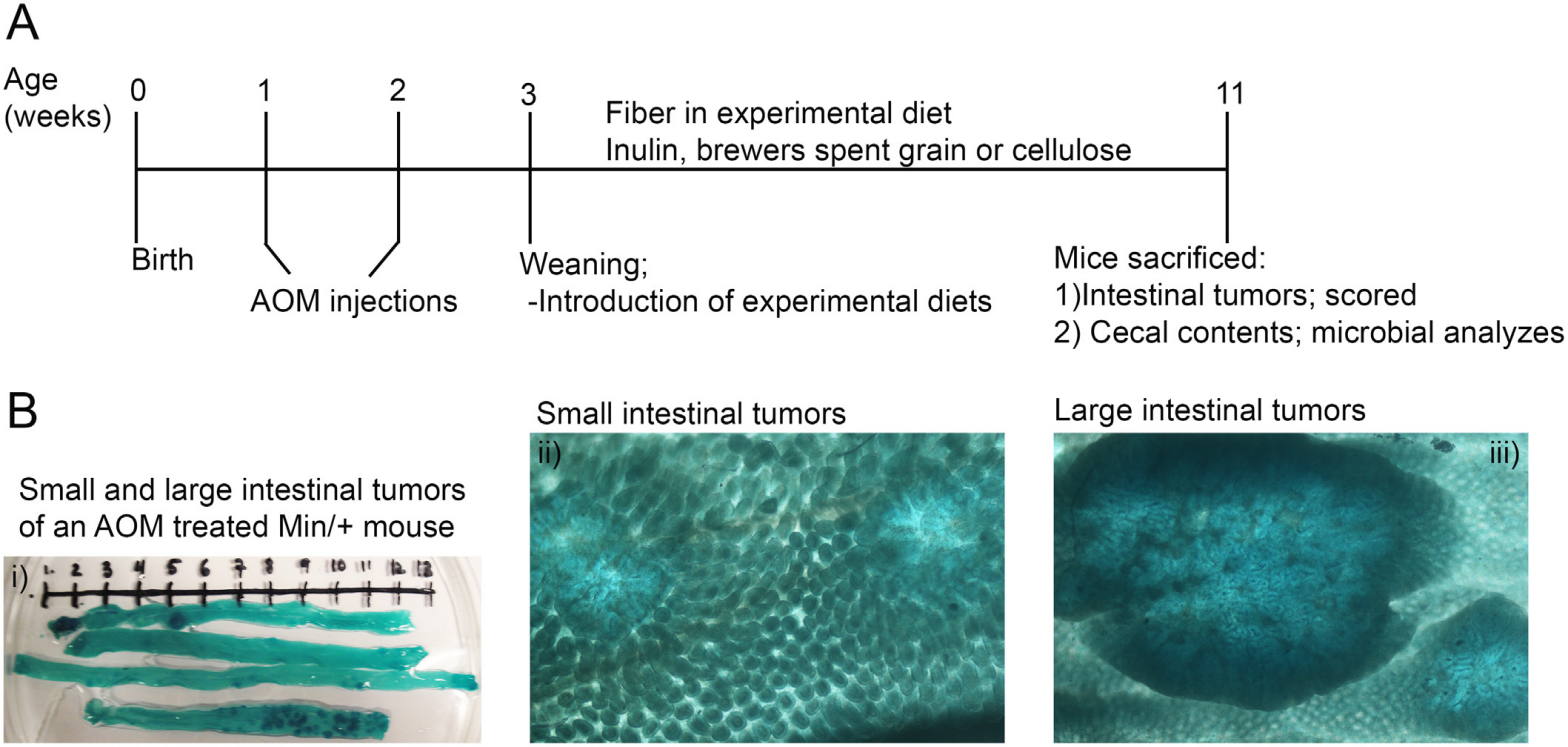
Experimental setup. **(A)** Timeline. A/J Min/+ mice were injected with azoxymethane (AOM) first one week after birth and secondly one week thereafter. Experimental diets were introduced at three weeks of age, and the mice were fed these diets until they were sacrificed at the age of eleven weeks. The intestines were prepared to score tumors and cecal contents collected for microbial analyzes. **(B)** Intestines from an AOM treated A/J Min/+ mouse stained in 0.2% methylene blue. Tumors were predominantly formed in the colon (i). Example of the tumor morphology of the small (ii) and large intestinal tumors (iii).

Diets containing one of three different fibers; CE, IN or BSG in either a lower (5%) or higher concentration (15%) were prepared based on the AIN-93M diet by Special Diets Services (SDS) (SDS, England, delivered by Scanbur, Oslo, Norway). The cellulose fiber was commercially available insoluble cellulose powder milled to a particle size < 100 μm. BSG is a non standardized dried high fiber water insoluble waste product from the brewing industry milled to a particle size < 1mm. BSG contains insoluble fibers but also proteins and other minerals that might contribute to the energy and nutrient content of the diet [38]. A water soluble commercial long chain (chain length ≥ 23) inulin preparation was used (Orafti HPX, Beneo). The average caloric value of the fiber preparations were 20, 15 and 16 MJ/kg for BSG, CE and IN, respectively, based on standard calorimetric measurements for feed (Parr 1281 Bomb Calorimeter, Parr Instrument Company, Moline, IL). The pups were introduced to experimental diets at weaning (21 days ±2 days) and fed fiber modulated diets for a period of 8 weeks until sacrification (77±3 days) (Fig 1). Dietary treatment was assigned randomly, but litters from the same breeding designated different treatments. Breeding was continued until minimum eight mice were included in each group (see Table 1 for number of mice in the treatment groups). Cages from all treatment groups were represented at different localizations in the rack to avoid factors related to localization (e.g. lightning conditions) to affect the experiment.

**Table 1:** Number of mice fed the different experimental diets (CE, BSG or IN) at a dose level of 5% or 15%.

**Fiber type**	**CE**				**BSG**				**IN**			
**Fiber %**	5		15		5		15		5		15	
**Gender**	Female	Male	Female	Male	Female	Male	Female	Male	Female	Male	Female	Male
**Mice**	12	9	11	8	15	10	12	13	17	8	12	9

### Dietary consumption and measures of body weight

The weight of the animals was measured at 1,2,3,6 and 11 weeks. Dietary consumption was measured weekly for each cage for the entire experimental period for a selection of litters, while for a limited period for others (data not shown).

### Sample collection

The animals were sacrificed at the age of 11 weeks ±3 days by cervical dislocation. The intestines were removed and prepared as earlier described [39, 40], including fixation for at least 48 hours in 4% paraformaldehyde prior to a ten second stain in 0.2% methylene blue (George T. Gurr LTd., London, UK). Contents of the cecum were collected, snap frozen in liquid nitrogen and stored at -80°C.

The intestines were examined, and quantification of tumors was performed by use of an inverted light microscope as previously reported [39, 40]. Tumors were scored by number and size (mm^2^), and tumor load (number of tumors × area of tumors = total area of tumors) was used as a measure of tumorigenesis.

### DNA extraction

DNA was extracted from cecal contents (17–150 mg fresh weight) of 68 male mice by mechanical lysis (FastPrep 24; Matrix E (Medinor)) and QiaAmp DNA Stool kit using a modified protocol. Briefly, the cecal content was added to the lysis tube together with 500 μl buffer ASL. The samples were lysed in a FastPrep instrument for 40 seconds at 6 m/s, then centrifuged for five minutes at 14000 ×g and the supernatants were transferred to new tubes containing 900 μl buffer ASL. The manufacture’s protocol was followed from this point. The DNA concentration was measured using the Quant-iT Picogreen ds DNA with picogreen (Invitrogen, Life Technologies).

### Analysis of microbiota

PCR was performed in triplicates and paired end sequencing (2×150bp) was performed using the protocol presented in ref 41 [41]. Briefly, the V4 region of the 16S rRNA gene was amplified with region-specific primers that included the Illumina flowcell adapter sequences. The reverse amplification primer also contained a twelve base barcode sequence that supports pooling of different samples. Samples were purified with Ampure (Agencourt Bioscience Corporation) and quantified using the Quant-iT Picogreen ds DNA with picogreen before pooling. The sample pool was purified and quantified as described above, diluted to 4nM, and sequenced on a MiSeq (Illumina) following the protocol provided by Illumina. In addition to the experimental samples, the MiSeq run also contained a control library made from phiX Control v3 which, in this run, accounted for 10% of reads. The library quantification and sequencing were performed at Nofima. The MiSeq Control Software (MCS) version used was RTA 1.17.28.

The total number of reads was 17,450,387. The forward and reverse reads were joined in QIIME version 1.7.0, resulting in 12,882,489 reads. Next, the barcodes corresponding to the reads that failed to assemble were removed. The sequences were then demultiplexed in QIIME allowing zero barcode errors and a quality score of 30 (Q30) using the QIIME toolkit [42]. The total number of sequences written was 6,828,382 with a median sequence length of 253 bp. The median number of sequences per sample was 99,321 sequences (max 137,781; min 61,964). Reads were assigned to their respective bacterial taxonomy using two-step open-reference operational taxonomic unit (OTU) picking workflow [43]. Briefly, after sequences were demultiplexed and quality filtered, reads were first clustered with a reference database (the Greengenes database (gg_13_5)) pre-clustered at 97% identity. Second, reads that did not group with any sequences in the reference collection were clustered *de novo*. Clustering at 97% identity was carried out using the UCLUST algorithm [44]. Reads that did not match a reference sequence were discarded. Chimeric sequences were removed in QIIME using ChimeraSlayer. Singeltons were removed, resulting in 17916 OTUs. Of these, 89% were ‘novel’ (i.e. not found in the Greengenes database (gg_13_5). Prior to the statistical analysis only those that satisfied at least one of two criteria were kept: 1) more than 0.005% in 75% or more of the individuals in at least one intervention group, or 2) more than 0.005% in 50% or more of all individuals. In total 507 OTUs passed this filter, each of these represents a phylotype and may be a representative of a bacterial species.

### Statistical analyses

Alpha- and beta diversity analysis on the microbiota were done in QIIME. To obtain an equal number of sequences across samples, the amplicon OTU table was resampled to an even depth of 50,000 sequences per sample. Beta diversity analysis showed that the dietary groups were best separated by unweighted unifrac (data not shown). The further multivariate analysis was therefore done on scaled data (mean zero and standard deviation equal to one), in order to give equal weight to all OTUs regardless of abundance.

Two-way ANOVA was used to analyze the experimental effects on single responses such as body weight, tumor load and the *Bacteroides/Firmicutes* ratio. The experimental effects on total microbiota were analyzed by 50–50 MANOVA [45], which is based on Principal Component Analysis (PCA) [46] and handles multiple collinear responses. The method calculates overall sums-of-squares and p-values for each experimental factor. Rotation testing was used to compute adjusted single response p-values according to false discovery rates. The relationship between total microbiota and tumor load was analyzed by Partial Least Squares Regression (PLSR) [46], and validated by full cross-validation. Variable significances were calculated by the sMC method [47].

The statistical analyses were performed using Minitab (v17, Minitab, Inc.) and MATLAB (R2014b, The MathWorks Inc.) with the 50–50 MANOVA toolbox (http://www.langsrud.com/stat/program.htm).

## Results

### Overview of the key responses caused by dietary fiber intervention and gender

In AOM treated A/J Min/+ mice we tested the influence of dietary fiber type (IN, CE or BSG) at two dose levels (5% or 15%) and gender on intestinal tumorigenesis, cecal microbiota of the male mice, relative cecum weight and body weight. The experimental factors (fiber type, fiber dose, gender, and their interactions) affected the biological parameters in varying degree, as expressed by explained variance (Table 2). Significant effects of fiber type, fiber dose and gender was seen on tumor load in the colon, but not in the small intestine. The effects of fiber type on colonic tumor load were independent of fiber dose and gender (shown by the non-significant interactions). Body weight was mainly influenced by gender, and relative cecum weight by fiber type. The microbiota, only investigated in male mice, was mainly affected by fiber type but also to some degree by fiber dose. The significant interaction between fiber type and fiber dose means that the effect of fiber dose was not the same for each fiber type. For all the biological parameters the residuals account for a large proportion of the variation, meaning that significant amounts of variation are explained by other factors than diet and gender.

**Table 2:** Overview of the responses caused by the different experimental factors (ANOVA tables for all biological parameters). The responses are shown as explained variance (%).

Experimental factors	Body weight	Relative cecum weight	Tumor load (small intestine)	Tumor load (colon)	Microbiota
	Explained variance (%)	Explained variance (%)	Explained variance (%)	Explained variance (%)	Explained variance (%)
**Fiber type (A)**	2.4**	45.2***	0.6 (ns)	14.2***	28.8***
**Fiber dose (B)**	0.0 (ns)	14.5***	0.9 (ns)	12.7***	4.1***
**Gender (C)**	35.1***	0.0 (ns)	0.7 (ns)	10.7***	n/a
**A x B**	0.3 (ns)	19.8***	0.7 (ns)	1.5 (ns)	6.1***
**A x C**	0.4 (ns)	0.0 (ns)	0.2 (ns)	1.5 (ns)	n/a
**B x C**	0.0 (ns)	0.0 (ns)	0.2 (ns)	0.5 (ns)	n/a
**Residuals**	61.7	20.4	96.7	58.8	61.0

* p<0.05;

** p<0.01;

*** p<0.001;

ns = not significant at 5% level. The p-values were calculated by Two-way ANOVA for single responses, and by 50–50 MANOVA for the microbiota. The microbiota was only analyzed in male mice; thereby the effect of gender on microbiota could not be evaluated (n/a).

### Effects of dietary fiber intervention on intestinal tumorigenesis, body weight and relative cecum weight

**Fig 2:**
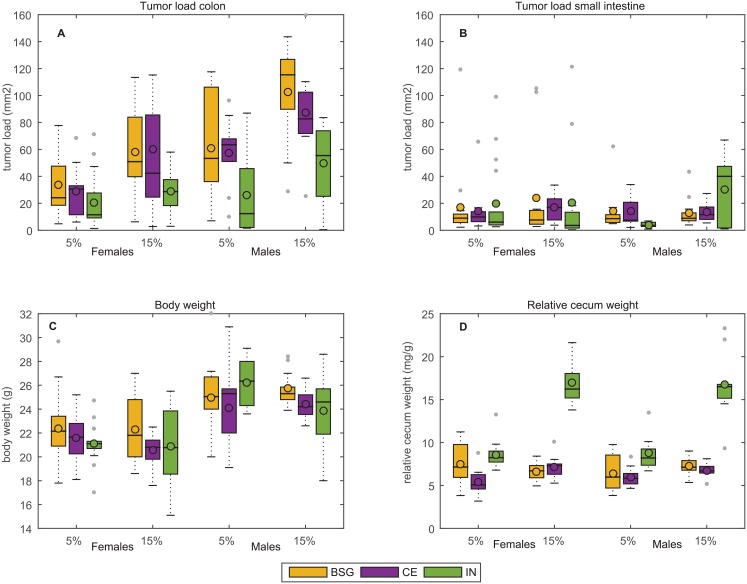
Boxplot showing differences between intervention groups. **(A)** Tumorigenesis in colon. **(B)** Tumorigenesis in small intestine. **(C)** Body weight. **(D)** Relative cecum weight. The fibers were tested at two concentrations (5% and 15%) in female and male A/J Min/+ mice treated with AOM. The * represents outliers (more/less than 3/2 times of the upper/lower quartile).

The tumor load was significantly higher in the colon than in the small intestine for all treatments (p<0.001) in the A/J Min/+ mice following AOM treatment (Table 2 and Fig 2). Small intestinal tumorigenesis was not found to be affected by diet or gender; effects were only observed on colonic tumorigenesis. Mice fed IN had significantly lower colonic tumor load than mice fed CE or BSG (p<0.001). This effect of IN on colonic tumor load, which was found for both fiber doses, was a result of both decreased tumor formation (number of tumors, p<0.001) and decreased tumor growth (size of tumors, p<0.001) (S1 Table). The distribution of tumors along the colonic anterior-posterior axis followed the same pattern for all groups (S1 Fig).

Interestingly all fibers demonstrated a dose-dependent stimulatory effect; in general the colonic tumor load increased 1.7 fold when the fiber concentration was elevated from 5% to 15% (p<0.001). Gender affected tumorigenesis as males had 1.7 times higher colonic tumor load than females (p<0.001). The relative cecum weight was significantly larger in mice fed IN than in mice fed CE or BSG (p<0.001); largest increase (2.4 fold) was seen with 15% fiber concentration. Enhanced relative cecum weight was seen with increased fiber concentration for IN (p<0.001) and CE (p<0.05). Males had larger body weight than females (p<0.001), and mice fed BSG had larger body weight than mice fed CE (p<0.1).

### Effects of dietary fiber intervention on cecal microbiota, and relationship between cecal microbiota and colonic tumor load

#### The cecal microbiota diversity was reduced in mice fed IN compared with mice fed CE or BSG

**Fig 3:**
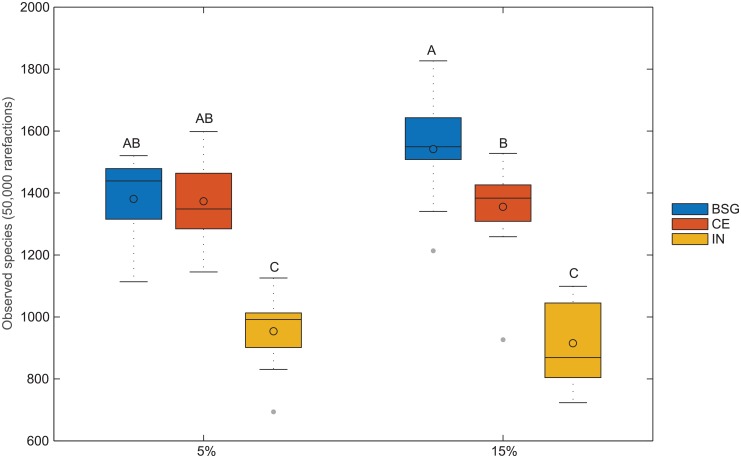
Effect of fiber type and fiber dose on the alpha diversity. Box plot of alpha diversity (observed species after 50,000 rarefactions). Grouping is according to the Turkey method (a single-step multiple comparison procedure and statistical test used to find means that are significantly different from each other). The different letters A, B and C are used to illustrate whether the mean difference between any pair of groups is statistically significant. Groups that do not share a letter are significantly different. The * represent outliers (less than 3/2 times of the lower quartile).

To investigate if the dietary intervention affected the bacterial diversity, we performed alpha diversity analysis (Fig 3). The number of observed species was significantly different between the three fiber groups (p<0.001), where IN fed mice had lowest number of observed species. The alpha diversity analysis also showed that IN had lower species richness (Chao1) and that the phylogenetic distance (PD_whole_tree) was lower for IN than BSG and CE (data not shown). At fiber dose of 15% the number of observed species was significantly different between the three fibers, with highest number of observed species in mice fed BSG>CE>IN.

#### The cecal microbiota composition was affected by the dietary intervention

**Fig 4:**
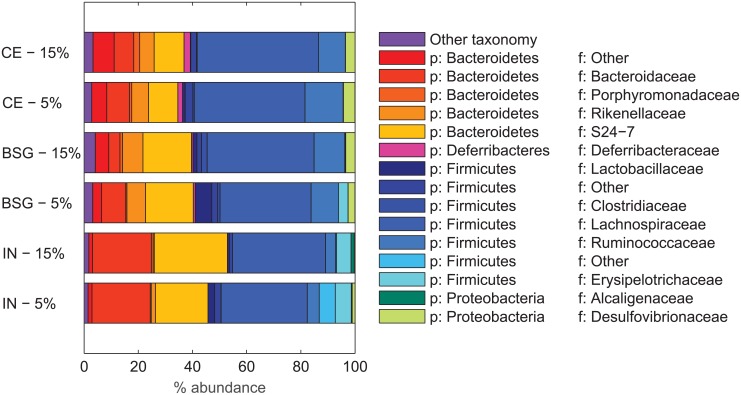
The different fibers’ effect on the composition of the cecal microbiota. The relative abundances of the cecal microbiota from mice in the different fiber groups were averaged. All families with average above 1% in at least one fiber group are represented. The remaining taxonomy is represented by “Other Taxonomy”. Phylum = p and family = f. The *Bacteroidetes* are represented by the red/orange colors while the *Firmicutes* are represented by the blue/turquoise colors.

The *Bacteroidetes/Firmicutes* ratios were significantly (p<0.005) different in mice fed the different fibers, with a higher mean value in IN (mean 1.0535) samples compared with CE (mean 0.7147) and BSG (mean 0.6455) (data not shown). Fig 4 illustrates the differences between the dominating phylum/families for the different fibers and doses.

IN enriched bacteria within the *Bacteroidaceae*, S24-7, *Erysipelotrichaceae* and *Alcaligenaceae* families. CE and BSG enriched bacteria within the *Rikenellaceae*, *Deferribacteriaceae* and *Desulfovibrionaceae* families.

**Fig 5:**
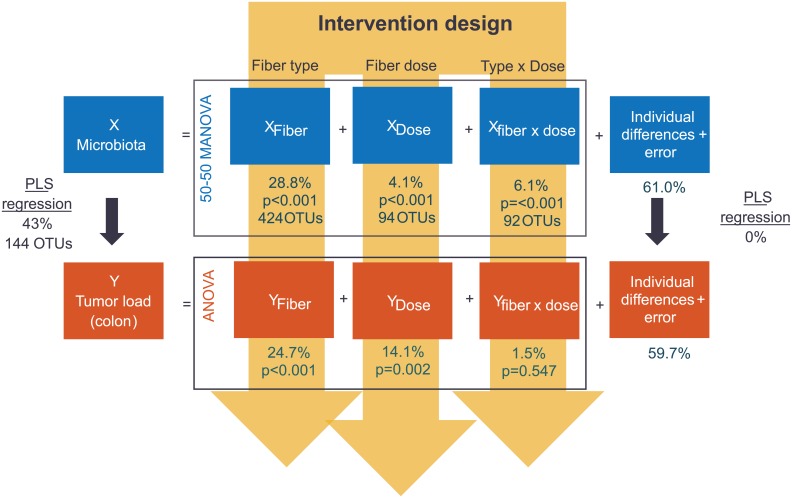
The joint analysis of microbiota and colon tumor load on male min mice, with overall results from Two-way ANOVA, 50–50 MANOVA and PLS regression. Note that the numbers for tumor load are different from Table 1, since this joint analysis was performed on male mice only. The numbers are % of explained variance and number of significant OTUs for each experimental factor and analysis method.

The microbiota was further evaluated in two ways: 1) with regard to the dietary treatments (50–50 MANOVA), and 2) with regard to the relation with colonic tumor load (PLS regression). The overall structure of these analyses is illustrated in Fig 5 (see also “Statistical analyses” under “Materials and Methods”).

**Fig 6:**
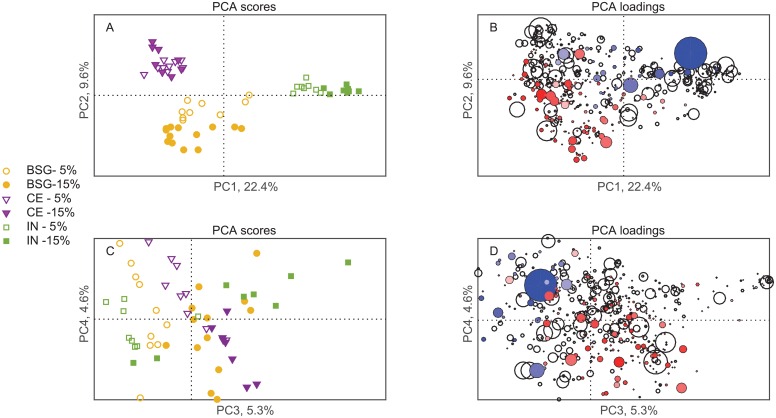
Scatter plots of scores and loadings from PCA on total microbiota. **(A)** Scores from PC1 and PC2. **(B)** Loadings from PC1 and PC2. **(C)** Scores from PC3 and PC4. **(D)** Loadings from PC3 and PC4. The samples in the score plots (A and C) are colored and marked according to fiber type and dose. The size of the loading plot bubbles (B and D) correspond to abundance of the OTUs, and the colored bubbles are those that were found to be significantly related to tumor load in the PLS regression: red = positive and blue = negative, and the color saturation corresponds to the magnitude of the correlation with tumor load. The largest bubble (blue) represent OTU 181719 (*Bacteroides*).

To illustrate the differences in cecal microbiota further, we performed PCA. Scores (the projection of samples on PCs) and loading (projection of OTUs on PCs) plots for the first four PCs are shown in Fig 6. A complete list of significant OTUs from 50–50 MANOVA and PLS regression are found in supplementary material (S2 Table).

The 50–50 MANOVA analysis showed that fiber type had a significant effect on 424 OTUs, and explained 28.8% of the overall OTU variation. The effects of fiber type on the microbiota is seen in Fig 6, where a clear separation of IN was seen along PC1, and a separation between CE and BSG along PC2. Since PC1 explains 27.6% and PC2 10.8% of the variance, this means that the difference between IN and CE/BSG was much larger than the difference between CE and BSG. The effect of dose only explained 4.1% (affected 94 OTUs) (Fig 5). However, the dose effect explained 14.1% of the variation in tumor load, which indicates that the differences in microbiota might be relevant, although small. A dose-proportional effect can be seen in the PCA plot for IN and BSG (Fig 6), with the low dose closer to the center of the plot. Note also that the variation within the BSG group is much larger than for IN and CE. PC3 and PC4 also shows some systematic differences, with a common dose-effect along PC3 (explaining 5.4%) and a tendency to fiber-dependent groupings within each dose level along PC4 (explaining 4.3%). The interaction between fiber and dose was also small, with a significant effect on 92 OTUs and explaining 6.1% of the variation. The interaction effect was non-significant for tumor load, indicating that the fiber-dependent dose effect found in the microbiota was not related to tumor load.

**Fig 7:**
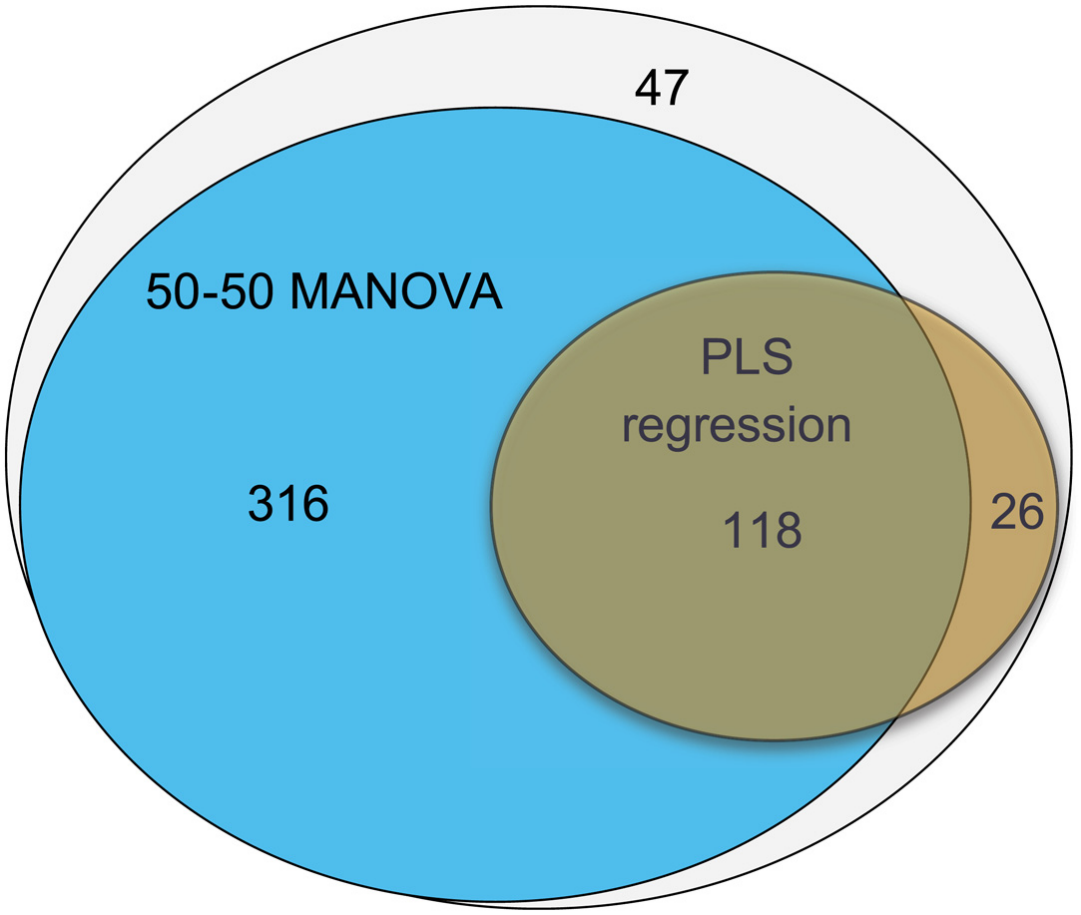
Venn diagram showing the overlap between OTUs identified by 50–50 MANOVA and PLS regression. The different statistical analysis was performed on 507 OTUs that passed the initial filtration. The 50–50 MANOVA identified 434 OTUs of which 118 OTUs overlapped with the 144 OTUs identified by the PLS regression. Twenty six OTUs of the OTUs identified from the PLS regression were not significant according to the dietary intervention.

**Fig 8:**
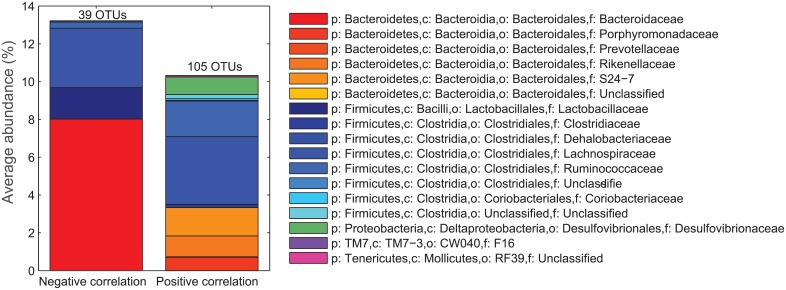
Overview (family level) of the OTUs (144) that were found to have significant relation to tumor load in the PLS regression. The negatively correlated OTUs are the 39 blue bubbles in the loading plots of Fig 6, while the 105 positively correlated OTUs are the red bubbles. Phylum = p; class = c; order = o and family = f.

The relationship between microbiota and colonic tumor load in male mice was analyzed with PLS regression without taking the intervention design into account. A model with 2 PLS-components explained 43% (cross-validated) of the variation in tumor load, and identified 144 OTUs with a significant relation to colonic tumor load (Fig 5). The majority of these OTUs correlated with the OTUs affected by the dietary fibers, and the overlap between OTUs identified by 50–50 MANOVA and PLS regression is shown in Fig 7 (Venn diagram). An overview of the dominating families within the negatively and positively correlated OTUs is given in Fig 8.

The 39 OTUs that were negatively related to tumor load were dominated by *Bacteroides* (within the *Bacteroidaceae* family) (see S1 Table for details). One of the dominating OTU (OTU 181719; *Bacteroides*) was also significantly enriched in mice fed IN (Fig 7 and S1 Table). *Bacteroides* was not represented among the OTUs positively related to tumor load (S1 Table), which were OTUs affiliated within the *Porphyromonadaceae* (genus *Parabacteroides*), *Prevotellaceae* (genus *Prevotella*), *Rikenellaceae*, S24-7, *Clostridiaceae*, *Dehalobacteriaceae* (genus *Dehalobacterium*), *Lachnospiraceae* (incl. genus *Dorea*), *Ruminococcaceae* (incl. genus *Oscillospira*), *Coriobacteriaceae* (genus *Adlercreutzia*) and *Desulfovibrionaceae* (incl. genus *Desulfovibrio*). The OTUs affiliated with S24-7 and positively related to tumor load was not the same as the S24-7 OTUs that were abundant in mice fed IN. Many OTUs affiliated to the *Lachnospiraceae* family were either positive or negative correlated to tumor load, demonstrating the complexity of this family.

PLS regression analysis of the residuals from the 50-50-MANOVA analysis revealed that individual differences between mice, independent of diet, were not related to tumor load (Fig 5) suggesting that the natural variation in microbiota did not cause any differences in the formation and growth of tumors. The analysis did, however, identify 26 OTUs that were significantly correlated to tumor load but not to our intervention (i.e. fiber, dose or interaction) (Fig 7). Among these 26 OTUs were representatives of *Lactobacillaceae* and a closer look on this group revealed that the correlation was caused by high abundance in a few individuals with low tumor load.

## Discussion

Our main findings were that the three different fibers affected the colonic tumor load differently. IN caused a tumor suppressive effect compared with the other fibers in AOM treated A/J Min/+ mice. Surprisingly, all three fibers induced a dose-dependent increase in colonic tumor load. Small intestinal tumor load was not affected by the dietary fiber interventions. We also identified a distinct cecal microbiota profile in the IN fed mice compared with CE and BSG fed mice. This profile was associated with low colonic tumor load. Analyzes also showed that the natural variation in microbiota was not related to differences in the colonic tumor load.

The influence of inulin in colonic carcinogenesis is equivocal, as previous studies have reported both procarcinogenic and anticarcinogenic effects. This lack of conformity may be explained by large variability in the experimental setups. Tumor formation and growth may be influenced by a number of factors in the experimental setup, e.g. type of rodent (mice or rat), strain (known to affect both frequency and location in mice), use of genetically modified (e.g. Min/+ mice) or wild type mice and the use of carcinogens. Moreover, variations are also found for fiber concentration, control diets, time for intervention and treatment duration. The inulin chain length may also be determinant, but are not always given. In addition, variability of microbiota composition between these models could be expected. Thereby most studies cannot directly be compared, and we refer to the reviews of Pool-Zobel et al. [48] and Roberfroid et al. [49] for a more in depth discussion of inulin and intestinal carcinogenesis. Briefly, in models applying AOM, the AOM precursor dimethyl hydrazine (DMH) or AOM/dextran sodium sulfate (DSS) to induce carcinogenesis, anti-tumorigenic effects of inulin have been found in mice [21, 50] and rats when administered prior and subsequent to the carcinogen [51–55]. In conventional C57BL/6J Min/+ mice, inulin has been reported to increase the genetically driven small intestinal tumor burden [56–58] except for one study reporting short chain FOS (which can be produced by degradation of inulin) to attenuate tumorigenesis [59]. These data may suggest a different role of fructose based fiber (inulin and FOS) in the genetically driven small intestinal tumorigenesis in C57BL/6J Min mice compared with carcinogen induced colon tumorigenesis. We did not find significant differences in the small intestinal tumor load between fiber types or dose in the AOM treated A/J Min/+ mice, but a significant reduced colonic tumor load in the IN fed mice. These data support the assumption of inulin primarily interfering with intestinal colonic carcinogenesis, possibly through gut microbiota fiber fermentation, and that inulin has minimal influence in the small intestine.

The higher tumor load at 15% fiber than 5% across all fiber types in our study is highly interesting as it may suggest that fiber stimulates carcinogenesis at a certain dose level. Previous studies with lower fiber levels showed increasing tumor attenuating effects of inulin at doses from 2.5 to 10% in AOM treated rats [53] and a tumor reductive effect of 6% inulin, but not 2%, was found in AOM/DSS treated BALB/c mice [21]. Although differences in the experimental setups, these data suggest that increasing the fiber level have beneficial effects on colonic cancer up to a certain level before it at higher fiber levels culminates.

As mentioned, it is reasonable to suggest that inulin has a minimal influence on the physicochemical environment of the small intestine like viscosity and water binding. It rather induces indirect effects in the gut by stimulation of certain bacteria and is extensively fermented [13] resulting in SCFA production, including butyrate [55, 60–62], associated with anti-tumorigenic effects *in vivo*. Our present work and studies performed by others have identified increased cecal weight following inulin treatment [48, 54, 63], probably as a result of increased microbial fermentation.

Accumulating data suggest that the microbiota has a role in the aetiology of several types of cancer by influencing inflammation, DNA damage and apoptosis [17, 18] and that the microbiota may drive tumorigenesis [20]. Our data showed a significant differentiation of the cecal microbiota between mice fed the different fibers, and that mice fed IN had lower species richness and a significantly higher *Bacteroidetes*/*Firmicutes* ratio compared with the other groups. High microbial diversity has been thought to be associated with a healthy gut, and low microbial diversity with disease [64]. However, the opposite was suggested in a metagenome-wide association study on stools from advanced adenoma and carcinoma patients and from healthy subjects [65]. They proposed that greater richness in genes or genera were not a sign of a healthy gut microbiota, but likely indicated overgrowth of a variety of harmful bacteria or archaea in patients with advanced colorectal adenoma or carcinoma. The lower species richness may also be correlated to the higher bioavailability of readily fermentable carbohydrates in IN compared with the other fibers, which is thought to favor only a few species with fast growth rate [66]. High nutrient availability has also been shown to reduce the diversity of the equine cecal microbiota [67]. Whether a microbiota with lower diversity is less resilient to environmental challenges and is less “healthier” for the host is not yet known [68]. In contrast, BSG contains a more complex fiber matrix, where it is likely that a large variety of bacteria needs to cooperate in order to digest this complex fiber. Cellulose on the other hand is an insoluble fiber known to undergo limited fermentation in the colon, but despite that, cellulose has been shown to substantially modulate the microbiota composition [69, 70]. Furthermore, cellulose supplementation ameliorates DSS induced colitis in mice [70].

Many of the OTUs that were enriched in mice fed IN were also associated with low tumor load. Among these were OTUs affiliated with the *Bacteroides* genus. A member of this genus, *Bacteroides uniformis*, has been identified as one of the main user of ^13^C-labeled inulin in rats [71]. *Bacteroides* have previously been both positively and negatively linked to CRC. Zhu et al. [72] observed that the phylum *Bacteroidetes* was less abundant in the lumen of CRC rats. A significant reduction of *Bacteroidetes* has also been reported in inflammatory bowel diseases such as Crohn’s disease and ulcerative colitis, which are known risk factors for CRC [73, 74]. On the other hand Feng et al. [65] reported that *Bacteroides* were overrepresented in carcinoma patients. Whether an observed dysbiosis represents a response to tumorigenesis or whether it precede tumor formation has been unclear. The genetic mutation of the A/J Min/+ mice may have a potential role on the microbiota and must be taken into account when interpreting the changes in the microbiota. A recent study has revealed that the mutation of the *Apc* gene alters the microbial interactions with the host intestinal mucosa prior to the development of polyposis [75]. They found that 6 week old C57Bl/6 Apc Min/+ mice with no detectable intestinal neoplasia exhibited an increased relative abundance of *Bacteroidetes* spp in the colon. They also found that the predominant family within the *Bacteroidetes* phylum was *S24*-*7*, whose relative abundance was also increased in the C57Bl/6 Apc Min/+ mice. In our study we found that IN increased members within the *Bacteroidetes* phylum, including *S24*-*7*. However, our results showed that there were many OTUs representing *S24*-*7*, and that the OTUs that correlated with increased tumor load were not the same OTUs that were increased in the mice fed IN. This illustrates the complexity of this family and that one must be careful to draw conclusions at this taxonomic level. Previous studies have also suggested that different *Bacteroides* strains may influence the health of the host through their colitogenic or probiotic potential [72]. Since we observed an increased abundance of *Bacteroides* in mice fed IN compared to CE or BSG, we argue that this increase is related to the fermentation of IN in the cecum and not to their Apc germline mutation. The association between IN induced microbiota and low tumor load should also be further investigated as it is possible that IN acts on both tumorigenesis and microbiota independently. To elucidate the potential tumorigenesis protective role of IN induced microbiota, e.g. *Bacteroides*, further studies are needed such as IN cecal or fecal microbiota transplant to germ-free or antibiotic treated A/J Min/+ mice.

Among the OTUs related to high tumor load were OTUs affiliated with the *Oscillospira* genera in *Ruminococcaceae* and the *Desulfovibrio* genera in *Desulfovibrionaceae* (Fig 8). *Oscillospira* has previously been related diet and found in higher prevalence in the stool of Native Africans than in African Americans [76], possibly related to the higher need of degradation of starch, hemicellulose and xylan in the Native African diet [76]. Specialist sulphate-reducing bacteria related to *Desulfovibrio* spp. are detectable in low numbers in most individuals [77]. Sulphide is toxic to colonocytes and inhibits butyrate oxidation, which may results in the breakdown of the colonocyte barrier [78]. Higher stool sulphide levels have been detected in patients with CRC compared with healthy controls, but so far an increase in *Desulfovibrio* spp. has not been found in fecal samples from patients with CRC in the few conducted studies [79].

In summary, our study in AOM treated A/J Min/+ mice indicates that fiber may play a pivotal role in the development of CRC. By comparing the effects of dietary interventions with IN, CE and BSG it was evident that IN suppressed colonic tumorigenesis. The IN fed mice displayed a distinct cecal microbiota profile that was associated with low colonic tumor load. Further studies are needed to clarify potential causal relationships between cecal microbiota and CRC.

## Supporting Information

S1 Fig Distribution of tumors along the colonic posterior-anterior axis. The distribution of colonic tumorload (mm^2^) along the posterior-anterior axis (cm from anus). (**A**) 15% fiber. (**B**) 5% fiber. The distribution of colonic tumorload (total area of tumors) along the posterior-anterior axis followed the same pattern for all fiber types in AOM treated A/J mice.
(TIF)

S1 Table Effects of dietary fiber intervention on colon tumorigenesis, body weight and relative cecum weight. The table contains information about mouse no (column A), gender (column B), treatment (column C), animal weight (column D), cecum weight (column E), number of tumors (column F), size of tumors (column G), tumor load (column H) and colon length (column I).
(XLSX)

S2 Table OTU table with results from statistical analyses. The table contains a list of all OTUs with OTU number and taxonomy (columns A-B). Results from the MANOVA model are p-values for the factors “Fiber type”, Fiber dose” and interaction “Fiber type x Fiber dose” (columns C-E), and average value for each fiber and dose combination (columns F-K). Column L contains sMC values from the PLS regression model, where OTUs with a sMC value above the critical value (cell Q2) are considered to have a significant correlation to the tumor load. The actual correlation between each OTU and tumor load is given in column M. In columns L-M the OTUs with a significant positive or negative correlation are colored red and blue respectively.
(XLSX)
