# Supplementary figures and images for: Effect of Dietary Fibers on Cecal Microbiota and Intestinal Tumorigenesis in Azoxymethane Treated A/J Min/+ Mice
Source: PLoS One. 2016 May 19;11(5):e0155402. doi: 10.1371/journal.pone.0155402 (PMC4873001; doi:10.1371/journal.pone.0155402)

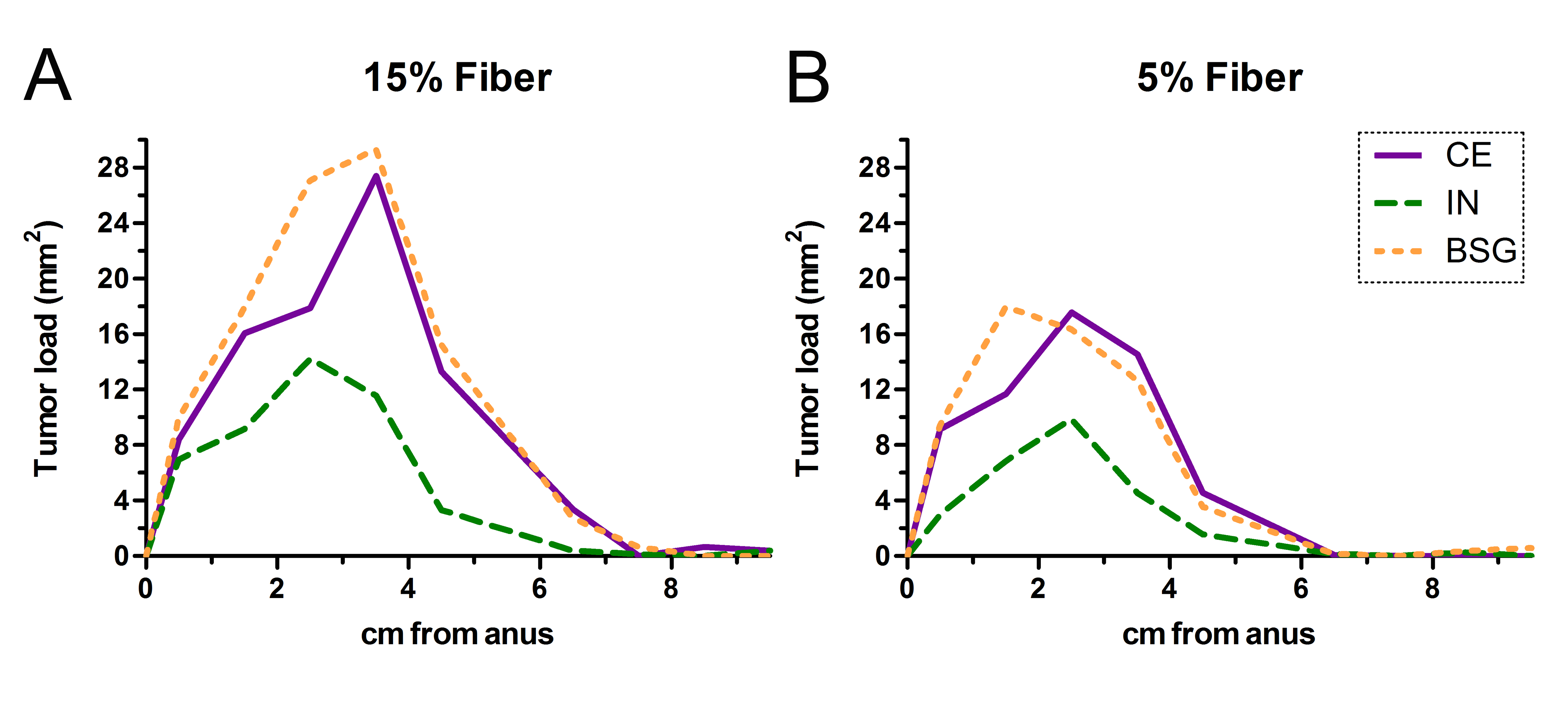

Supplement: S1 Fig — The distribution of colonic tumorload (mm2) along the posterior-anterior axis (cm from anus). (A) 15% fiber. (B) 5% fiber. The distribution of colonic tumorload (total area of tumors) along the posterior-anterior axis followed the same pattern for all fiber types in AOM treated A/J mice. (TIF) [file pone.0155402.s001.tif]
